# Items and Selections

**Published:** 1872-09

**Authors:** E. N. Brush


ITEMS AND SELECTIONS. *By E. N. Brush.*—Dr. Edwin Payne, M. R. C. P. Lond., (*London Lancet,*) speaks of the use of Hydrastis Canadensis as pleasant and painless lotion in Cancer. The strength in which it was used was a drachm of the tincture to eight ounces of water.——Dr. R. J. Levis reports in the *Philadelphia Medical Times* a successful case of treatment of Aneurism of the external iliac by complete compression. Total arrest of circulation was affected, and continued during profound anæsthesia by ether, for five hours and a half. The patient showing at this time some signs of exhaustion, the compression was discontinued. Slight pulsation continued until the seventh day, when it was evident that pulsation had ceased and that the tumor was much shrunken The author draws the following conclusions: 1. That aneurism of the external iliac artery may be amenable to treatment by complete compression of the vessel in a brief period. 2. That total arrest of pulsation can be effectually made by mechanical means. 3. That compression of the external iliac at the cardiac extremity of the aneurism, probably even where aneurismal dilatation exists at the seat of pressure, and without the aid of aortic compression, may be sufficient for the cure. 4. That anæsthesia is essential to such treatment, and that prolonged etherization does not prevent coagulation of the blood. 5. That pulsation may not cease entirely for some days, even when a coagulum has been fully formed, 6. That reduction in size of the aneurismal tumor, by shrinking of the clot, is more rapid and complete when cured by total compression than when the cure is effected by slow deposit of fibrinous laminæ in the gradual or partial compression. 7. That the treatment of aneurism of the external iliac by the metnod of complete compression is the safest and most reliable, and should be generally adopted."——Dr. M. N. Miller in the *Medical Record* reports two cases Mydriasis, which were accompanied by gastric and intestinal irritation, and which were cured by removing these troubles. The trouble in one case was obstinate constipation, the dilitation of the pupil being relieved after active purgation. The other case yielded to treatment directed to intestinal parasites.——The Publishers of the *Philadelphia Medical Times* propose to issue their Journal weeklv instead of twice a month as heretofore. To meet the additional expense the price has been advanced to five dollars per year.——We learn from the *Record* that a death from ether has recently occurred in the Bellevue Hospital.

Dr. Julian J. Chisolm of Baltimore reports in the *American Journal of Medical Sciences* a complete removal of the iris during a fight by the finger nail of an antagonist without any injury to the sight.——Dr. Samuel C. Bussey of Washington, D. C., reports (*American Journal of Obstetrics*) three cases of obliteration of the Cervex Uteri which have come under his observation, to support the views of Prof. Isaac Taylor of New York.——Dr. G. H. Bixby of Boston (*Journal of the Gynæcological Society*) reports the successful removal of an ovarian tumor weighing not far from thirty pounds from a woman aged forty-five. The operation was delayed half an hour, after some of the cysts had been brought to light and emptied, by the threatening aspect of the patient. After the injection of two ounces of brandy into the rectum and the application of the poles of a galvanic battery to the palms of the hands, the patient rallied and the operation was proceeded with.

Eight weeks after the operation the patient performed a journey of more than one hundred miles in the cars.

We can not but notice the extreme caution displayed by the surgeon to have everything on hand which might be needed by the patient, or during the course of the operation. He divides the preparations into two heads. 1st. the duties of the friends at the house, under which he enumerates the preparation of some thirty three different articles for the use of the patient.

2nd. The duties of the surgeon. Under this head he enumerates in order some forty-three different articles for the use of the surgeon and his assistants.

Dr. Lowenberg of Berlin, advances a new and ingenious Method of removing foreign bodies from the ear. The tip of a pencil of charpie is dipped into some joiners glue; the pencil is then carried into the ear carefully until it rests slightly against the foreign substance. The patient is cautioned to remain quiet for from three quarters of an hour to an hour, when the pencil may be removed bringing with it the foreign substance.——We notice that a recent discussion in the Buffalo Medical Association in regard to Cerebro-Spinal Meningitis in which its characteristics, name, treatment, etc., were some what fully discussed, has attracted attention in England and is spoken of in *The Doctor* one of our most excellent exchanges.—A very interesting clinical lecture on Lithotomy by Prof. Humphry, F. R. S. is published in the *Lancet* for September. In speaking of the instruments he gives the following excellent advice: "It is advisable to adhere to a few instruments, just as it is advisable to adhere to a few remedies. The man who uses a few weapons is more likely to wield them skilfully than he who tries, and so perplexes himself with many."

The *Washington Evening Star* in commenting upon the mortality among the children as shown by the recent mortality tables prepared by Dr. J. M. Toner uses the following language:

"In view of the appalling proportion of infant mortality, especially in our large cities, shown by the statistics above referred to, Dr. Toner some time ago called attention to the necessity for adopting some general measures for checking this vast waste of infant life. We have various humane institutions, insane asylums, &c., to alleviate the sufferings and lengthen out the lives of the wrecks of humanity, but we have literally nothing to stay the immense waste of life of the hopeful young, full of great promise. It is certainly better to provide for the preservation of the lives of those who will grow into usefulness, than to confine all our humane efforts to guarding the health of the hopeless shattered wrecks."

Prof. Parvin, one of the editors of the American Practitioner has resigned his position as Prof. of Medical and Surgical Diseases of Women and Children in the University of Louisville.——Professor Linton for many years the chief editor of the St. Louis Medical Journal and its originator died at St. Louis on the 2nd of June, Dr. Linton graduated in 1835 and has been in the active practice of medicine since that time.

He for many years held the position of Professor of the Principles and Practice of Medicine in St. Louis Medical College.

Prof. Linton had been in ill health for some time and his decease although it will be somewhat of a shock to his distant friends had been expected for some time by his immediate circle of acquaintences.

